# High-Throughput Sequencing Reveals Previously Undetected Viruses and Mixed Infections in Pepper (*Capsicum annuum*) in Hungary

**DOI:** 10.3390/v18040481

**Published:** 2026-04-21

**Authors:** Emese Demián, Réka Sáray, Asztéria Almási, Kata Pogácsás, Katalin Salánki

**Affiliations:** 1 Department of Plant Pathology, Plant Protection Institute, Centre for Agricultural Research, Hungarian Research Network (HUN-REN), 1116 Budapest, Hungary; demian.emese@atk.hun-ren.hu (E.D.); saray.reka@atk.hun-ren.hu (R.S.); almasi.aszteria@atk.hun-ren.hu (A.A.); 2 Horticultural Plant Genetics Group, Department of Plant Biotechnology, Institute of Genetics and Biotechnology, Hungarian University of Agriculture and Life Sciences, 1118 Budapest, Hungary; pogi.kataa@gmail.com

**Keywords:** *Capsicum annuum*, high-throughput sequencing, pepper virome, eggplant mottled dwarf virus, tobacco vein clearing virus, endogenous pararetrovirus, persistent viruses

## Abstract

The increasing global movement of plant material and the complexity of viral communities associated with cultivated crops complicate routine plant virus diagnostics. High-throughput sequencing (HTS) has therefore become an important tool for the comprehensive characterization of plant viromes. In this study, symptomatic pepper (*Capsicum annuum*) samples submitted to our laboratory between 2020 and 2025 were investigated using HTS following unsuccessful routine diagnostic assays, despite the presence of virus-like symptoms. Virome analysis revealed the presence of multiple viruses with distinct biological characteristics. Eggplant mottled dwarf virus (EMDV) sequences were identified, representing, to our knowledge, the first sequence data from Hungary. In addition, sequences related to tobacco vein clearing virus (TVCV) showed highest similarity to endogenous viral element present in *Capsicum annuum* genome assemblies. Persistent viruses, including bell pepper alphaendornavirus (BPEV) and pepper cryptic virus 2 (PCV2), were also detected. These findings demonstrate the complex viral communities associated with cultivated pepper and highlight the limitations of strictly targeted diagnostic approaches. The results emphasize the value of HTS for comprehensive virome characterization in horticultural crops.

## 1. Introduction

The European Union’s accumulated fresh market pepper (*Capsicum annuum* L.) harvest volume increased to 2.995 million tons in 2014, an increase of 5.5% compared to 2.84 million tons in 2023. The area under cultivation also expanded from 55.6 thousand hectares in 2023 to 58.1 thousand hectares, indicating the importance of pepper breeding in the European Union. The volume of pepper production in Hungary ranks 6th place in fresh market pepper with 96,700 tons and 4th place with 138,000 tons in protected cultivation pepper production, indicating its significance in the Hungarian vegetable cultivation [1].

Significant pests and diseases in pepper cultivation are powdery mildew, downy mildew and true bugs, which requires continuous plant protection intervention. From a plant protection perspective, aphids and mites caused the most damage in greenhouses and plastic tents in recent years which consequently increased the impact of viral infections on pepper production. Outbreaks of viral pandemics can result in severe losses in pepper breeding by reducing the yield and the quality of the production. Several plant viruses were reported to infect pepper. In 2003, their number was estimated to be 68 [2] which significantly increased over the last years, and recently it was estimated to exceed 165 viruses [3]. For decades, the most frequent and significant viral pathogens of pepper in Hungary were tobacco mosaic virus, (TMV; species: *Tobamovirus tabaci*), tomato mosaic virus (ToMV; species: *Tobamovirus tomatotessellati*), pepper mild mottle virus (PMMoV; species: *Tobamovirus capsici*), tomato spotted wilt virus (TSWV; species: *Orthotospovirus tomatomaculae*), cucumber mosaic virus (CMV; species: *Cucumovirus CMV*) and potato virus Y (PVY; *Potyvirus yituberosi*) [4].

These viruses are well-characterized and have been extensively studied due to their economic importance. They can induce diverse symptoms and frequently occur in mixed infections, complicating diagnosis and disease management [5,6,7].

Although these viruses represent the most significant causes of economic losses in pepper, emerging viruses over the past decade have also had a substantial impact on pepper breeding in Europe due to increased seed and seedling transport, as well as climate-driven changes in vector populations. In addition, mixed infections involving less well-characterized viruses may significantly affect pepper quality.

Eggplant mottled dwarf virus (EMDV; species: *Alphanucleorhabdovirus melongenae*) is a negative-sense RNA virus belonging to the family *Rhabdoviridae* [8] and infecting several solanaceous crops in Europe [9]. It is transmitted by leafhoppers and has been associated with symptoms such as vein yellowing, chlorosis, leaf curling, growth reduction and fruit mottling [10]. Based on publicly available sequence databases and the EPPO Global Database [11], no EMDV data originating from Hungary is currently available.

Tobacco vein clearing virus (TVCV; species: *Solendovirus venanicotianae*) is a member of the genus *Solendovirus* in the family *Caulimoviridae* [12]. Sequences related to TVCV have been identified in several solanaceous hosts, including *Solanum lycopersicum* and *C. annuum*, where they may represent endogenous pararetroviral elements (EPRVs) integrated into the host genome [13,14]. As caulimoviral sequences can occur either as integrated genomic elements or as episomal viral genomes, sequence-based analyses alone may not allow clear distinction between these forms [14].

Bell pepper alphaendornavirus (BPEV; species: *Alphaendornavirus capsici*) is the member of the genus *Alphaendornavirus* in the family *Endornaviridae* [15]. Members of this family are persistent single-stranded, positive-sense RNA viruses that are predominantly transmitted vertically through seed [15,16]. BPEV has been detected in pepper and is considered a symptomless virus, as infected and virus-free plants are phenotypically indistinguishable, even in the presence of co-infecting pathogenic viruses [17].

Pepper cryptic virus 2 (PCV2; species: *Deltapartitivirus duocapsici*) is the member of the genus *Deltapartitivirus* in the family *Partitiviridae* [18]. Members of this family are bisegmented double-stranded RNA viruses that establish persistent infections in plants and are predominantly transmitted through seed [18,19]. PCV2 has been detected in pepper and is considered a symptomless virus, with no consistent association with visible disease symptoms [20,21].

A multiplex RT-PCR assay was developed previously to detect the most economically important pepper-infecting viruses with adequate sensitivity. This assay is able to detect both CMV subgroups, tobamoviruses, PVY and TSWV [22]. It has been routinely and successfully applied in our laboratory for virus detection in protected cultivation systems in Hungary. In most cases, the assay proved to be sufficiently sensitive; however, in some pepper samples, no viruses were detected despite the presence of typical virus-like symptoms. In the present study, five pepper samples were selected in which either no virus was identified by the multiplex RT-PCR assay, or the detected virus did not correspond to the observed symptom phenotype. To further investigate these unresolved cases, high-throughput sequencing (HTS) was applied as an exploratory approach to characterize the virome composition of these plants.

## 2. Materials and Methods

### 2.1. Plant Material and Sample Preparation

**Figure 1:**
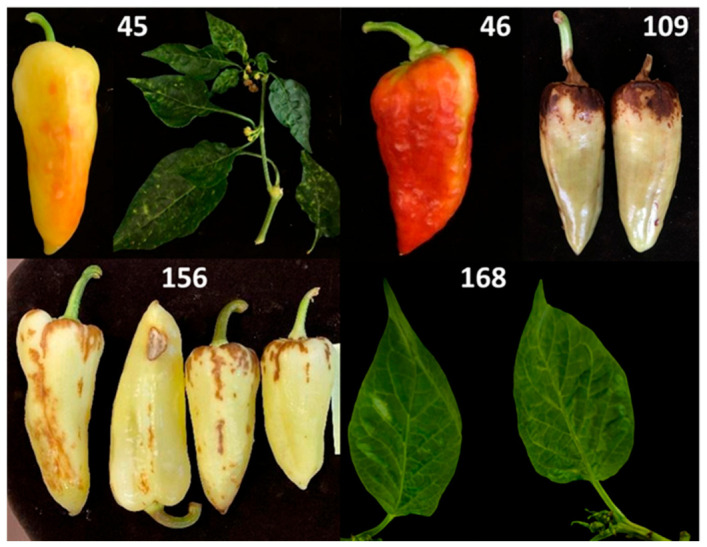
Representative fruits and leaves of *C. annuum* samples used in this study. Fruits from samples 45, 46, 109 and 156 and leaves from samples 45 and 168 are shown. Sample identifiers correspond to those used throughout the study. Observed symptoms are shown for descriptive purposes only.

Five *C. annuum* plants showing virus-like symptoms were selected for analysis (Figure 1). The pepper samples originated from greenhouse-grown production in Szentes, Hungary. All samples were submitted by the grower and received as symptomatic plant material. The samples analysed in this study were collected between 2020 and 2025.

### 2.2. RNA Extraction and High-Throughput Sequencing (HTS)

Total RNA was extracted individually from each plant sample using the Direct-zol™ RNA Miniprep Kit (Zymo Research, Irvine, CA, USA) according to the manufacturer’s instructions. The extraction protocol includes an on-column DNase treatment, resulting in DNA-free RNA of sufficient quality and quantity for downstream applications. RNA concentration and quality were assessed prior to pooling.

Equal amounts of total RNA from each of the five plant samples were combined to generate a pooled RNA sample for sequencing. Pooling of RNA prior to HTS limits sample-level resolution and may produce composite sequence assemblies representing mixed viral populations. Library preparation using a ribosomal RNA-depleted long non-coding RNA-sequencing protocol and HTS were carried out by Novogene Europe (Munich, Germany) on an Illumina NovaSeq X Plus platform, generating 150 bp paired-end reads.

The raw RNA-seq dataset consisted of 22,121,392 paired-end reads. Raw RNA-seq data supporting the findings of this study are available in the NCBI Sequence Read Archive (SRA) under BioProject accession PRJNA1405328 (Table S1).

### 2.3. Bioinformatics Analysis and Virus Identification

Quality control, read processing, and downstream bioinformatics analyses were performed using Geneious Prime (version 2025.2.2). De novo assembly of the RNA-seq reads was conducted within Geneious Prime using the Geneious de novo assembler with default parameters. To ensure stable assembly performance in accordance with the software recommendations, the maximum number of contigs retained per assembly was limited to 1000.

Initial sequencing and assembly statistics, including the total number of reads, the number of non-redundant reads, and the number of contigs generated, are summarized in Table S1.

Assembled contigs were screened for viral sequences using BLASTn searches implemented in Geneious Prime (version 2025.2.2) against a locally constructed NCBI RefSeq viral genome database. BLASTn hits were ranked based on E-value, and contigs showing an E-value of 0 were considered preliminary positive viral candidates. Identified viral sequences were further evaluated based on sequence similarity to known plant viruses.

Genome coverage for the detected viruses was calculated by mapping non-redundant RNA-seq reads to the corresponding viral reference genomes. Coverage statistics for the analysed viruses are summarized in Table S2. For EMDV, virus-specific contigs were mapped to a reference genome, enabling reconstruction of the complete viral genome sequence.

### 2.4. RT-PCR Validation, Cloning, and Sanger Sequencing

To validate virus detection and to determine the distribution of individual viruses among the sampled plants, complementary DNA (cDNA) synthesis was performed both from the pooled RNA sample used for HTS and from RNA extracted separately from each of the five plants.

First-strand cDNA was synthesized using the RevertAid First Strand cDNA Synthesis Kit (Thermo Fisher Scientific, Waltham, MA, USA) with random hexamer primers, according to the manufacturer’s protocol. The cDNA derived from the pooled RNA sample served to confirm HTS results, whereas cDNA generated from individual plant RNAs was used to identify which viruses were present in each plant.

PCR amplification was carried out using virus-specific primer pairs either adopted from the literature or newly designed based on contig sequences obtained from RNA-seq data (Table S3). Amplifications were performed with Phire Hot Start II DNA Polymerase (Thermo Fisher Scientific, Waltham, MA, USA). Primer-specific annealing temperatures were optimized and are listed in Table S3.

Amplified DNA fragments were separated by agarose gel electrophoresis, and products of the expected size were excised and purified using the GeneJET Gel Extraction Kit (Thermo Fisher Scientific, Waltham, MA, USA). Purified PCR products were cloned into pJET1.2/blunt cloning vector using the CloneJET PCR Cloning Kit (Thermo Fisher Scientific, Waltham, MA, USA). Recombinant clones were subjected to Sanger sequencing (Eurofins Biomi Ltd., Gödöllő, Hungary).

### 2.5. Phylogenetic Analysis

Phylogenetic analyses were conducted for EMDV, BPEV, PCV2 and TVCV. Viral sequences used for tree construction and their corresponding GenBank accession numbers and genomic regions are listed in Table S5. Phylogenetic analyses were performed to determine the evolutionary relationships of the viral sequences identified in this study. Multiple sequence alignments were generated using reference sequences retrieved from the NCBI GenBank database. Phylogenetic trees were inferred using the Maximum Likelihood (ML) method implemented in MEGA 12 [23]. Phylogenetic trees were rooted using homologous sequences of closely related viruses as outgroups.

For each dataset, the best-fitting nucleotide substitution model was selected prior to tree reconstruction. The following models were applied: Tamura 3-parameter (T92) for the EMDV L1 region and the BPEV 7787–8473 nt region, Hasegawa–Kishino–Yano (HKY) for the BPEV 12,652–13,218 nt region, Tamura–Nei (TN93) for the TVCV coat protein sequences and Kimura 2-parameter (K2P) for the PCV2 RNA2 sequences. Evolutionary rate differences among sites were modelled using a discrete Gamma distribution where applicable.

The robustness of the inferred phylogenies was evaluated by bootstrap analysis with 1000 replicates. Initial tree topologies for heuristic searches were generated using Neighbor-Joining or Maximum Parsimony methods and subsequently optimized under the Maximum Likelihood criterion. Positions with less than 95% site coverage were excluded using partial deletion. The resulting phylogenetic trees were visualised and edited in MEGA 12 [23].

## 3. Results

### 3.1. Symptoms Observed in Sampled Pepper Plants

The analysed pepper samples exhibited various virus-like symptoms affecting both fruits and leaves. Fruit symptoms included discoloration, fruit surface roughness and necrotic spots, while leaves showed mosaic-like patterns and vein-associated chlorotic areas. Representative examples of symptomatic fruits and leaves from selected samples are shown in Figure 1.

### 3.2. HTS-Based Virome Characterization

To characterize the virome of symptomatic pepper plants, high-throughput RNA sequencing was performed on pooled RNA samples obtained from five individual plants. Bioinformatics analysis of the RNA-seq data started with quality assessment of the sequencing reads. The reads were suitable for downstream analyses, and no residual adapter sequences were detected. A summary of the initial sequencing statistics is provided in Table S1.

Non-redundant reads were assembled de novo, resulting in approximately 1000 contigs. These contigs were screened using a custom BLAST pipeline against a database of available viral reference genomes. This analysis identified contigs related to six virus-associated sequences, indicating the presence of multiple viral sequences in the analysed pooled sample. To further verify virus presence and genome coverage, non-redundant reads were mapped to the corresponding viral reference genomes. This allowed evaluation of read support and genome coverage for each virus. Detailed results are summarized in Table S2. Based on the combined results of de novo assembly, BLAST analysis, and read mapping, TSWV, PMMoV, PCV2, BPEV, EMDV and TVCV were considered to be present in the pooled pepper RNA sample.

Genome coverage analysis showed clear differences among the detected viruses. Complete genome coverage (100%) was obtained for EMDV, PMMoV, and all three genomic RNA segments of TSWV. BPEV showed near-complete genome coverage (99.8%). In contrast, TVCV showed partial genome coverage (68.1%), while PCV2 showed incomplete coverage, with 87.9% coverage for RNA1 and 53.3% for RNA2.

### 3.3. EMDV Genome Reconstruction

EMDV-specific contigs obtained by de novo assembly were used for reference-guided assembly with the GenBank reference genome of EMDV (accession no. NC_025389.1). This assembly resulted in the reconstruction of a near-complete consensus genome of EMDV. Due to the pooling strategy, this sequence should be interpreted as a consensus representation of the detected viral population rather than a strain-resolved genome from an individual plant. A majority-rule approach was used for consensus sequence generation, in which the nucleotide present at the highest frequency at each genomic position was selected. This approach considers sequence variation within the viral population.

To further confirm the robustness and read support of the HTS-derived EMDV genome, an additional consensus sequence was generated by mapping non-redundant reads to the EMDV reference genome. The two consensus sequences obtained from contig-based assembly and read mapping were compared and combined. As both approaches produced consistent results, a final complete EMDV genome sequence was reconstructed from the HTS data and used for further analyses. The complete genome sequence of the Hungarian EMDV variant has been deposited in the GenBank database under accession number PX926506.

### 3.4. RT-PCR Confirmation and Sequence Comparison of Detected Viruses

In total, six viruses were identified by HTS analysis and were subsequently examined by RT-PCR using cDNA derived from pooled and individual pepper samples. Virus-specific RT-PCR assays were performed to confirm the HTS results and to determine the distribution of the detected viruses among the five individual plants.

**Figure 2:**
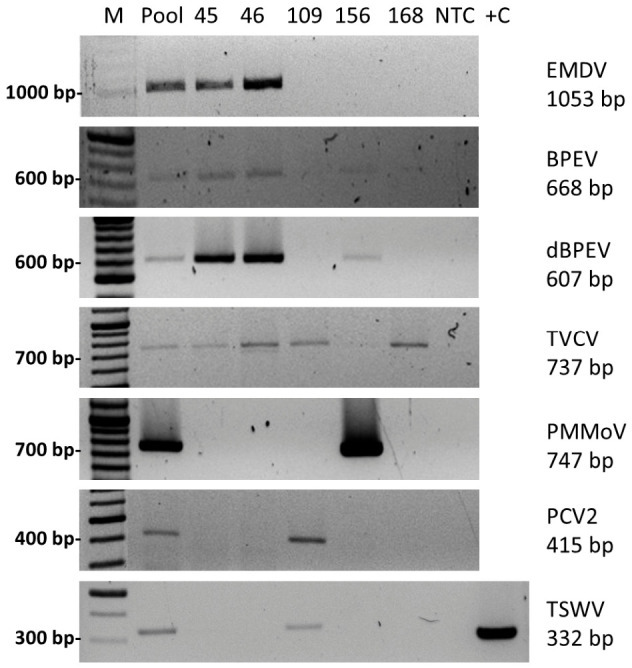
RT-PCR detection of identified plant viruses in *C. annuum* samples. Agarose gel electrophoresis images show amplification products obtained with virus-specific primer sets. A positive control (+C) was available only for TSWV. For the remaining assays, RNA pools previously shown by high-throughput sequencing (HTS) to contain the respective viral sequences were used as reference sample. The labels BPEV and dBPEV correspond to the primer pairs listed in Table S3, targeting different genomic regions and producing distinguishable PCR products. No-template controls (NTC) were included for each reaction and showed no amplification. Molecular weight markers (M) are indicated on the left.

All viruses identified by HTS data were successfully detected by RT-PCR in both pooled and individual plant samples. A summary of HTS- and RT-PCR-based virus detection is provided in Table S4, while representative RT-PCR results are shown in Figure 2.

Based on RT-PCR analyses, pepper plants numbered 45 and 46 were positive for EMDV, BPEV, and TVCV. TSWV, PCV2, and TVCV were identified in sample 109. Sample 156 was positive for PMMoV and BPEV. In sample 168, only TVCV was detected.

To further characterize the detected viruses, selected RT-PCR products were subjected to Sanger sequencing.

Partial L1 region sequences of EMDV obtained from samples 45 and 46 were deposited in GenBank under accession numbers PX704448 and PX704449. Partial coat protein gene sequences of TVCV were obtained and deposited in GenBank under accession numbers PX704442–PX704445. For BPEV, two regions of the polyprotein-coding sequence were sequenced. The 669 nt sequences corresponding to the region closer to the 5′ end of the genome were deposited in GenBank under accession numbers PX704436–PX704438, while the 567 nt sequences encompassing the RdRp region were deposited under accession numbers PX704439–PX704441. The partial RNA2 sequence of PCV2 obtained in this study was deposited in GenBank under accession number PX704446.

The resulting nucleotide sequences were compared with reference genomes and available sequences in the GenBank database using BLASTn analysis. A summary of nucleotide identities to reference genomes and to the closest related variants is provided in Table S5.

The near-complete consensus EMDV genome shared 89% nucleotide identity with the reference genome and 93% identity with the closest available isolate in GenBank.

The TVCV coat protein sequences showed approximately 81% nucleotide identity to the TVCV reference genome and the highest identity (99%) to a sequence derived from a *C. annuum* genome assembly (GenBank accession no. KU997025.1). The presence of highly similar TVCV-like sequences within the pepper genome is consistent with previously reported endogenous pararetroviral elements in *Solanaceae* species.

The partial PCV2 RNA2 sequence showed high nucleotide identity (99%) to previously reported PCV2 isolates. The BPEV sequences shared 85–88% nucleotide identity with the reference genome and up to 99% identity with selected GenBank isolates, depending on the genomic region analysed.

### 3.5. Phylogenetic Analysis of Detected Viruses

Phylogenetic analyses were performed for EMDV, TVCV, BPEV and PCV2 to determine the genetic relationships of the detected variants with previously reported sequences.

#### 3.5.1. Phylogenetic Analysis of EMDV

EMDV was detected in two individual pepper plants (samples 45 and 46). Partial Sanger sequencing of the L1 region revealed that the two sequences were 100% identical at the nucleotide level.

**Figure 3:**
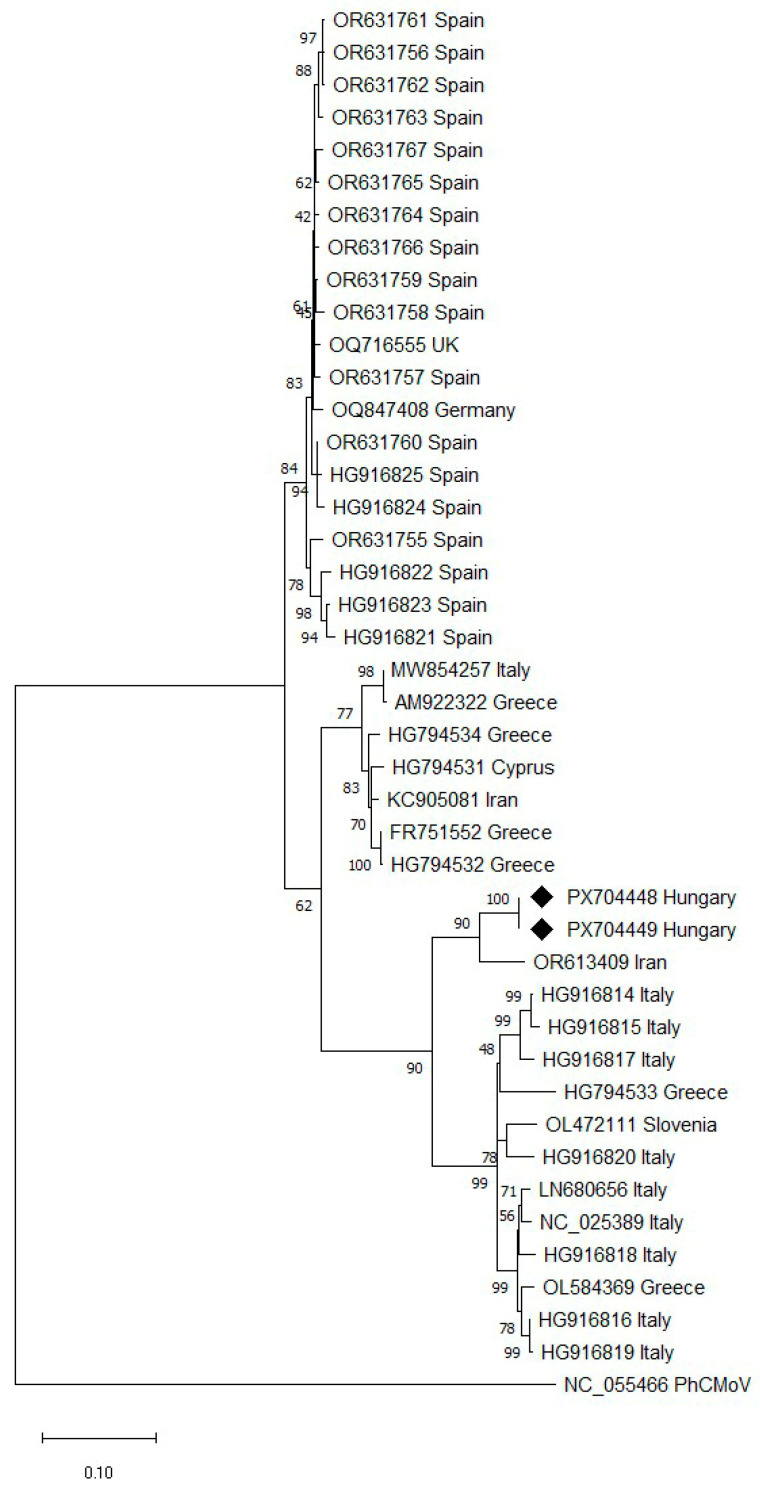
Maximum likelihood phylogenetic tree based on the partial EMDV L gene (L1 region) obtained by Sanger sequencing. The tree was constructed using the best-fitting nucleotide substitution model in MEGA 12. Bootstrap values (1000 replicates) greater than 50% are shown next to the branches. The tree was rooted using physostegia chlorotic mottle virus (PhCMoV; NC_055466.1) as an outgroup. The sequences generated in this study (PX704448 and PX704449) are indicated with black diamonds. Geographic origin is shown next to sequence accession numbers.

To evaluate the phylogenetic position of the Hungarian EMDV variant, phylogenetic analysis was performed based on the partial L1 region obtained by Sanger sequencing (Figure 3). The Hungarian variants (PX704448, PX704449) clustered with an Iranian isolate (OR613409), supported by high bootstrap values, within a broader European–Middle Eastern lineage.

#### 3.5.2. Phylogenetic Analysis of TVCV

For phylogenetic analysis, the Sanger-derived CP sequences were subjected to BLASTn searches, and representative TVCV-like sequences derived from *Solanaceae* genome assemblies were selected (Table S6).

**Figure 4:**
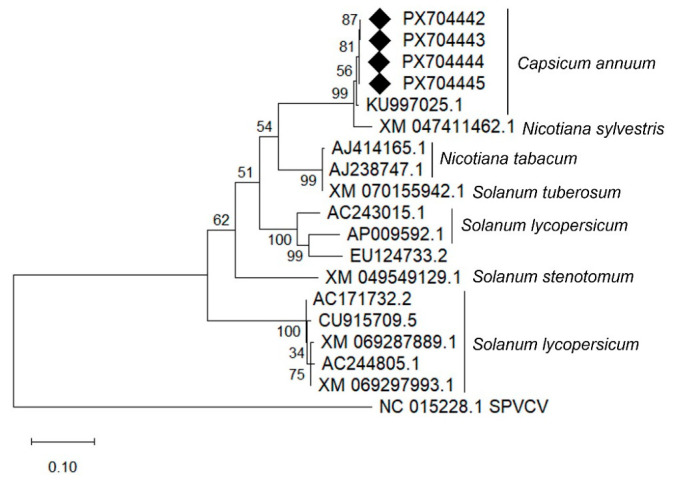
Maximum likelihood phylogenetic tree of TVCV nucleotide sequences constructed using the best-fit substitution model in MEGA 12. Bootstrap support values based on 1000 replicates are indicated at the nodes. The scale bar represents the number of nucleotide substitutions per site. The tree was rooted using sweet potato vein clearing virus (SPVCV; NC_015228.1) as an outgroup. Sequences generated in this study are indicated with black diamonds. The remaining sequences represent genome-derived TVCV-like elements identified in different *Solanaceae* species. Detailed information on sequences included in the analysis is provided in Table S6.

In the phylogenetic tree, the four Hungarian TVCV sequences clustered in a well-supported group (bootstrap value 100). This cluster grouped with virus-like sequences derived from *C. annuum* genome assemblies (Figure 4).

#### 3.5.3. Phylogenetic Analysis of BPEV

Two regions of the BPEV polyprotein-coding sequence were used for phylogenetic analysis. The first region corresponded to nucleotide positions 7787–8473 of the reference genome, while the second region spanned positions 12,652–13,218 and included RdRp domain of the polyprotein.

**Figure 5:**
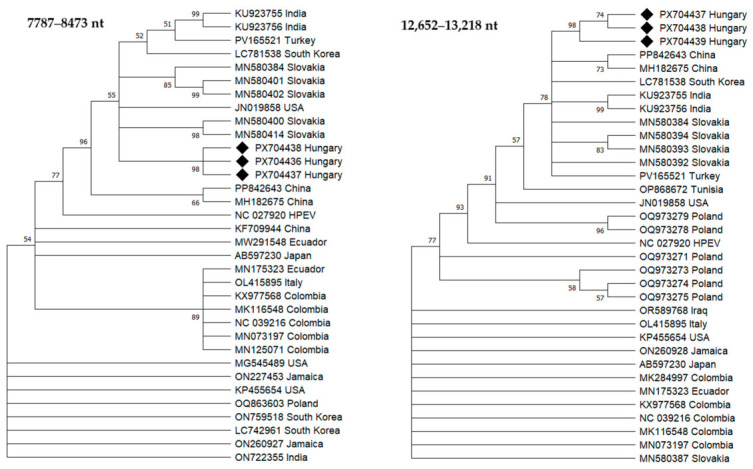
Maximum likelihood phylogenetic trees constructed from partial BPEV sequences encompassing the 7787–8473 nt and 12,652–13,218 nt regions based on the reference genome (NC_039216). The trees were constructed using the best-fitting nucleotide substitution model in MEGA 12. Bootstrap values (1000 replicates) greater than 50% are shown next to the branches. The trees were rooted using hot pepper endornavirus (HPEV; NC_027920.1) as an outgroup. The sequences generated in this study (PX704436-PX704441) are indicated with black diamonds. Geographic origin is shown next to sequence accession numbers.

Phylogenetic analyses based on both polyprotein regions (nt 7787–8473 and nt 12,652–13,218) showed that the Hungarian BPEV variants formed a distinct cluster supported by high bootstrap values (Figure 5). In the phylogenetic tree constructed from the RdRp region (nt 12,652–13,218), the Hungarian variants clustered with BPEV sequences originating from Slovakia. In contrast, in the tree based on the nt 7787–8473 region, the Hungarian sequences grouped with isolates reported from India, Turkey, and South Korea (Figure 5).

#### 3.5.4. Phylogenetic Analysis of PCV2

HTS analysis identified a contig corresponding to RNA1 of PCV2, which encodes the RdRp. In contrast, no contig matching RNA2 of PCV2, which encodes the CP, was detected by the HTS analysis when mapped to the reference genome.

For phylogenetic analysis, BLASTn searches were performed, and PCV2 RNA2 sequences available in the GenBank database and covering the full length of the amplified region (375 nt) were selected. The partial PCV2 RNA2 sequence obtained in this study was included in the phylogenetic analysis.

**Figure 6:**
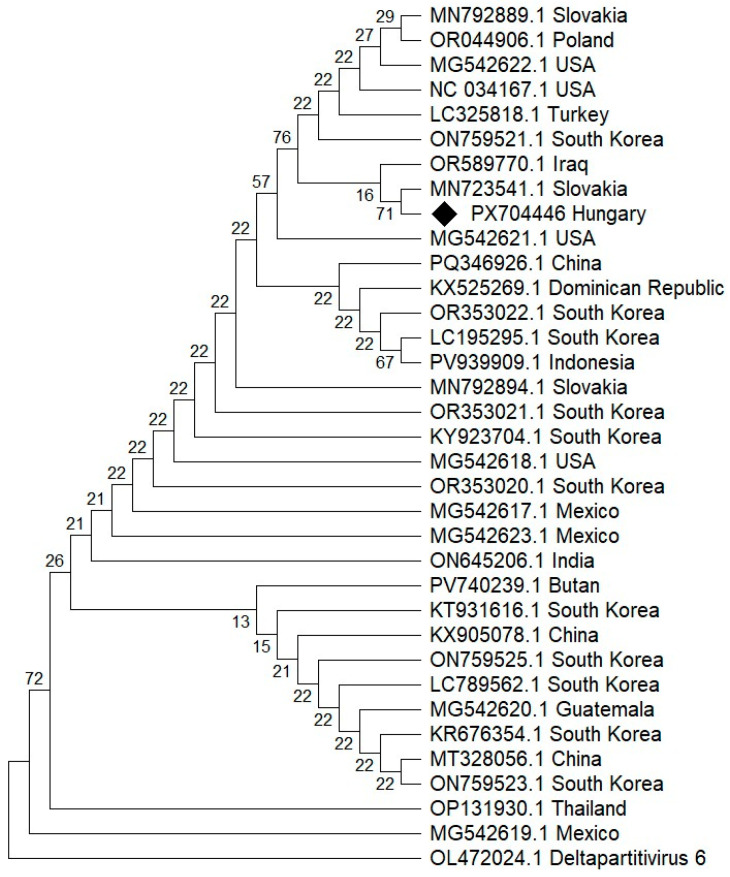
Maximum likelihood phylogenetic tree of PCV2 nucleotide sequences inferred using the best-fit nucleotide substitution model (Kimura 2-parameter) determined by model selection in MEGA 12. Bootstrap values based on 1000 replicates are shown at the nodes. The scale bar indicates the number of nucleotide substitutions per site. The tree was rooted using plant-associated deltapartitivirus 6 (OL472024.1) as an outgroup. GenBank accession numbers and countries of origin are indicated for each sequence. The sequence generated in this study (PX704446) is indicated with a black diamond. Geographic origin is shown next to sequence accession numbers.

In the phylogenetic tree based on partial PCV2 RNA2 sequences, the Hungarian variant (PX704446) clustered with PCV2 isolates originating from Slovakia, Iraq, and Turkey. This grouping was supported by a bootstrap value of 76 (Figure 6).

## 4. Discussion

In Hungary, economically important pepper viruses are routinely monitored using targeted molecular diagnostic approaches, including RT-PCR-based assays directed against well-established viruses [22]. These approaches are highly effective for the detection of known high-impact viruses and form the backbone of phytosanitary and certification workflows in commercial pepper production. At the same time, the increasing international movement of seeds and planting material has facilitated the long-distance dissemination of several viruses infecting *C. annuum* [5,24]. Under these conditions, diagnostic strategies restricted to predefined target viruses may overlook emerging, less characterized, or persistent viral pathogens.

Between 2020 and 2025, several symptomatic pepper samples submitted to our laboratory yielded either negative results in routine diagnostics or resulted in virus identifications that did not fully clarify the observed symptoms. These findings highlighted the limitations of strictly targeted approaches and led to the application of non-targeted HTS. This method provided a comprehensive overview of the pepper virome and enabled the detection of viruses that are not typically identified by targeted diagnostic assays.

Pooling of samples prior to HTS limits sample-level resolution and prevents direct association between detected viruses and individual plant symptoms. Therefore, HTS results should be interpreted at the virome level. Although subsequent RT-PCR and Sanger sequencing performed on individual plant samples enabled targeted validation of virus presence and assessment of their distribution among plants, no causal relationship between detected viruses and observed symptoms can be established without fulfilling Koch’s postulates.

Mixed viral infections are more and more common in cultivated crops, leading to important epidemiological and evolutionary consequences [25]. In intensively managed horticultural systems, vegetative propagation, seed transmission, and the movement of plant material between regions can promote the persistence and spread of multiple viruses within crop populations [26]. As a consequence, plants often harbour diverse viral communities that include both potentially pathogenic viruses and persistent viruses, which typically form long-term, frequently symptomless associations with their hosts [27]. Such complexity further underscores the limitations of strictly targeted diagnostic methods in comprehensive virome assessment [28].

### 4.1. Eggplant Mottled Dwarf Virus in Hungary

To our knowledge, this study provides the first confirmed detection of EMDV in Hungary. A near-complete consensus genome of EMDV was reconstructed from HTS data and deposited in GenBank. Due to the pooling strategy, this sequence should be interpreted as a consensus representation of the detected viral population. According to the EPPO Global Database, EMDV has been reported from several countries in Europe, North and West Africa, and Western Asia, where it infects solanaceous crops as well as a range of ornamental and wild plant species [11]. The identification of EMDV in Hungarian pepper production systems therefore extends the documented distribution of the virus within Central Europe.

Phylogenetic analysis based on the Sanger-derived L1 region showed that Hungarian EMDV variants clustered with an Iranian isolate (OR613409), showing the highest nucleotide identity. Since phylogenetic proximity alone does not allow inference of the geographic origin or route of introduction of the virus, the origin of EMDV in Hungary is still unknown.

EMDV is typically transmitted in a persistent, propagative manner by leafhoppers [29,30]. Seed transmission has not been reported [9], and mechanical transmission has been demonstrated only under experimental conditions with limited efficiency [31]. Consequently, long-distance dissemination is most likely associated with the movement of infected plant material or viruliferous insect vectors.

Several faunistic surveys confirm the presence of *Anaceratagallia* species in Hungary, including agricultural environments such as apple, pear, and apricot orchards. In particular, *A. ribauti* and *A. laevis* have been documented in Hungarian agroecosystems several times [32,33,34]. The confirmed presence of these species in cultivated landscapes suggests that ecological conditions compatible with EMDV vector populations are present in Hungary. However, the vector competence of Hungarian populations for EMDV has not yet been experimentally verified.

In addition, EMDV infects a wide range of cultivated, ornamental, and wild plant species, including several vegetatively propagated ornamentals and documented weed hosts [9]. The movement of infected propagative material, particularly ornamental plants, may therefore represent a plausible pathway for virus introduction. Furthermore, the presence of alternative reservoir hosts in agroecosystems could facilitate local virus persistence if competent vectors are present. However, the relative contribution of these potential pathways to the introduction and establishment of EMDV in Hungary cannot be determined based on the currently available data.

### 4.2. Tobacco Vein Clearing Virus-Related Sequences in Pepper

TVCV was detected in four out of five symptomatic pepper plants by HTS and subsequently confirmed by RT-PCR using primers designed based on the assembled contigs. The consistent detection of TVCV-related sequences in multiple samples supports their recurrent detection in the analysed pepper plants.

BLASTn analysis revealed that the closest matches to the identified TVCV sequences frequently corresponded to sequence data derived from *C. annuum* genome assemblies rather than from annotated viral genomes. In most cases, these sequences were not explicitly annotated as virus-like elements. This pattern is consistent with the known occurrence of members of the family *Caulimoviridae* as EPRVs integrated into plant genomes [11]. Indeed, TVCV-related EPRVs have previously been reported in multiple solanaceous hosts, including *Nicotiana* spp., *S. lycopersicum*, *C. annuum* and *S. melongena*, based on genomic and PCR-based analyses [13,14,35].

TVCV has not been demonstrated to be transmissible by aphids or mechanical inoculation, and infection in *N. edwardsonii* is believed to originate from the activation of replication-competent endogenous TVCV (eTVCV) sequences integrated into the host genome. Thus, documented cases of TVCV infection are closely associated with endogenous viral elements rather than with conventional horizontal transmission [35]. The apparent discrepancy between this restricted and activation-based infection model and the broader distribution of TVCV-related sequences in *Solanaceae* genomes complicates the biological interpretation of RNA-based detection. In this context, the detection of TVCV-related RNA sequences in pepper raises questions regarding the biological status of these elements. While RNA detection and independent RT-PCR confirmation indicate transcriptional activity, the available data do not allow discrimination between episomal viral infection and transcription of genome-integrated viral sequences.

As previously mentioned, TVCV-related sequences were identified in *C. annuum* through genomic and PCR-based approaches [14]. However, these data were interpreted in the context of EPRVs embedded in the host genome. The high similarity of the sequences identified in our study to TVCV-related sequences present in publicly available pepper genome datasets further supports the widespread occurrence of such endogenous elements in *Solanaceae* species. At the same time, the detection of TVCV-related RNA in multiple independent samples highlights the importance of careful interpretation of HTS data when identifying pararetroviruses and other virus groups capable of genome integration.

In one sample (168), TVCV was detected in the absence of other viruses, providing an opportunity for further observation, although no causal relationship can be established. This pepper plant initially exhibited reduced growth and mild vein clearing (Figure 1). Vein clearing and growth retardation have been described as characteristic symptoms associated with activation of endogenous TVCV in *N. edwardsonii* [35]. However, in the present case, symptoms gradually disappeared over a three-month period, and the plant subsequently showed normal development. Such transient symptom expression differs from the more persistent symptomatology described in the case of *N. edwardsonii* infection [35] and may reflect processes that cannot be resolved based on the available data.

Taken together, the available data do not allow a clear distinction between episomal viral infection and transcriptionally active endogenous viral elements and therefore the biological relevance of the detected TVCV-related sequences remains uncertain.

### 4.3. Persistent Pepper Viruses in Hungary

Persistent viruses belonging to the families *Partitiviridae* and *Endornaviridae* were also identified in the analysed pepper samples. PCV2 and BPEV were detected by HTS and confirmed by RT-PCR in several individual plants. In contrast to acute plant viruses, members of these virus groups are typically transmitted vertically, often persist in plants without inducing obvious symptoms, and are generally not associated with clear disease phenotypes [21]. Consequently, their biological characterization relies primarily on molecular and phylogenetic analyses rather than on symptom-based diagnostics.

PCV2 was originally described from the cultivar ‘Hungarian Wax’; however, this designation refers to the pepper variety and does not necessarily indicate a Hungarian geographical origin of the sequenced isolates [20]. The original study was carried out in the United States therefore the analysed plant material likely originated from locally cultivated plants. To our knowledge, sequence-based characterization of Hungarian PCV2 isolates has not been available to date. Based on our search of public sequence databases, no PCV2 sequence data derived from *C. annuum* reported from Hungary are currently accessible in GenBank.

Similarly, BPEV is a persistent endornavirus of pepper that has been detected in multiple cultivars and is maintained through efficient vertical transmission without causing obvious symptoms [16]. To our knowledge, sequence data derived from Hungarian pepper isolates are not available to date. Therefore, the sequences identified in this study provide the first molecular characterization of Hungarian PCV2 and BPEV variants from pepper and contribute to the growing dataset required for population-level and phylogenetic analyses of persistent viruses.

Phylogenetic analyses demonstrated that the Hungarian PCV2 variant clustered with previously reported European and West Asian isolates, whereas the Hungarian BPEV variants formed a distinct cluster. Since persistent viruses are typically asymptomatic and efficiently transmitted through vertical inheritance [18], their biological characterization relies largely on sequence-based comparisons rather than on symptomatology. The generation of well-documented regional sequence data is therefore essential for assessing genetic diversity, geographic distribution, and evolutionary relationships of these viruses in cultivated pepper plants. These findings highlight that persistent viruses represent stable yet largely invisible components of the cultivated pepper virome. Their detection through non-targeted HTS underscores the value of comprehensive molecular approaches for uncovering long-term virus–host associations that may remain unnoticed in symptom-based surveys.

Overall, the combined detection of an emerging pathogenic virus (EMDV), transcriptionally active pararetrovirus-related sequences (TVCV), and persistent seed-transmitted viruses (PCV2 and BPEV) illustrates the complexity of viral communities that can be present in symptomatic pepper plants. These findings provide a limited, exploratory insight into the pepper virome based on the analysed samples. They also support that non-targeted HTS enables simultaneous identification of epidemiologically relevant pathogens and cryptic, vertically maintained viruses that would likely remain undetected using conventional diagnostic approaches. Such comprehensive virome-level analyses can contribute to a more complete understanding of virus diversity, transmission dynamics, and long-term virus–host associations in cultivated crops.

## 5. Conclusions

This study provides a near-complete consensus genome sequence of EMDV derived from pepper plants in Hungary, contributing new sequence data for this virus in publicly available databases. In addition, sequences related to TVCV were detected in pepper plants, most likely representing transcriptionally active endogenous pararetroviral elements integrated in the *C. annuum* genome. Persistent viruses, including BPEV and PCV2 were also identified and molecularly characterised from Hungarian pepper samples. Together, these findings illustrate the complex and multilayered structure of viral communities that may be present in symptomatic pepper plants, while reflecting a limited, sample-specific insight rather than a comprehensive overview of the pepper virome in Hungary. These results further illustrate how virome-level analyses can complement routine diagnostics and improve our understanding of virus diversity in agricultural systems. It should be noted that the conclusions of this study are based on a limited number of samples (*n* = 5) and therefore do not allow generalization to the broader pepper production system in Hungary.

## Data Availability

The raw sequencing data generated in this study are available in the NCBI Sequence Read Archive (SRA) under accession number SRR36895163, associated with BioProject PRJNA1405328 and BioSample SAMN54741365. The complete genome sequence of Eggplant mottled dwarf virus assembled from HTS data has been deposited in GenBank under accession number PX926506. Additional viral sequences obtained by Sanger sequencing have been deposited in GenBank under accession numbers PX704436–PX704449. Additional data supporting the findings of this study are available in the Supplementary Materials (Tables S1–S6).

## Supplementary Materials

The following supporting information can be downloaded at: https://www.mdpi.com/article/10.3390/v18040481/s1.; Table S1: Initial RNA-seq statistics; Table S2: Summary of the bioinformatic analysis; Table S3: Sequences of PCR primers used for virus detection; Table S4: Summary of viral detection results obtained by HTS and RT-PCR; Table S5: Summary of viral sequences deposited in NCBI GenBank; Table S6: Genome-derived TVCV-like sequences from Solanaceae species used for phylogenetic analysis.

## References

[1] FruitVeB Bulletin: Zöldség-Gyümölcs Ágazati Jelentés 2024. https://www.nak.hu/kiadvanyok/8441-fruitveb-bulletin-zoldseg-gyumolcs-agazati-jelentes-2024.

[2] Pernezny K., Roberts P.D., Murphy J.F., Goldberg N.P. (2003). Compendium of Pepper Diseases.

[3] Ali A. (2024). Overview of RNA Viruses Infecting Capsicum Species. Pepper Virome: Molecular Biology, Diagnostics and Management.

[4] Tóbiás I., Almási A., Csilléry G., Nemes K., Salánki K. (2017). Virus Diseases of Pepper (*Capsicum annuum* L.) in Hungary. Agric. Food.

[5] Jones R.A.C. (2021). Global Plant Virus Disease Pandemics and Epidemics. Plants.

[6] Pruss G., Ge X., Shi X.M., Carrington J.C., Bowman Vance V. (1997). Plant Viral Synergism: The Potyviral Genome Encodes a Broad-Range Pathogenicity Enhancer That Transactivates Replication of Heterologous Viruses. Plant Cell.

[7] German T.L., Ullman D.E., Moyer J.W. (1992). Tospoviruses: Diagnosis, Molecular Biology, Phylogeny, and Vector Relationships. Annu. Rev. Phytopathol..

[8] Walker P.J., Freitas-Astúa J., Bejerman N., Blasdell K.R., Breyta R., Dietzgen R.G., Fooks A.R., Kondo H., Kurath G., Kuzmin I.V. (2022). ICTV Virus Taxonomy Profile: Rhabdoviridae 2022. J. Gen. Virol..

[9] Pappi P.G., Maliogka V.I., Amoutzias G.D., Katis N.I. (2016). Genetic Variation of Eggplant Mottled Dwarf Virus from Annual and Perennial Plant Hosts. Arch. Virol..

[10] Roggero P., Milne R.G., Masenga V., Ogliara P., Stravato V.M. (1995). First Reports of Eggplant Mottled Dwarf Rhabdovirus in Cucumber and in Pepper. Plant Dis..

[11] European and Mediterranean Plant Protection Organization Eggplant Mottled Dwarf Virus (EMDV). https://gd.eppo.int.

[12] Teycheney P.-Y., Geering A.D.W., Dasgupta I., Hull R., Kreuze J.F., Lockhart B., Muller E., Olszewski N., Pappu H., Pooggin M.M. (2020). ICTV Virus Taxonomy Profile: Caulimoviridae. J. Gen. Virol..

[13] Othman Abass M., Lahuf A.A. (2023). High-Throughput Sequencing and Bioinformatic Analysis Reveal Presence of the Endogenous Pararetrovirus Tobacco Vein Clearing Virus Genome in the Tomato (*Solanum lycopersicum*) Host Genome. Arab. J. Plant Prot..

[14] Zitouna N., Sakka H., Bouteraa M.T., Mehrez M., Slatni T., Fakhfakh H., Ben Hamed K., Gorsane F. (2026). Genetic Diversity and Evolution of Endogenous Pararetroviruses across Solanaceae: How Farming Systems Drive Dynamic Tomato EPRVS Changes under Salt Stress. Front. Plant Sci..

[15] Valverde R.A., Khalifa M.E., Okada R., Fukuhara T., Sabanadzovic S., ICTV Report Consortium (2019). ICTV Virus Taxonomy Profile: Endornaviridae. J. Gen. Virol..

[16] Fukuhara T. (2019). Endornaviruses: Persistent dsRNA Viruses with Symbiotic Properties in Diverse Eukaryotes. Virus Genes.

[17] Okada R., Kiyota E., Sabanadzovic S., Moriyama H., Fukuhara T., Saha P., Roossinck M.J., Severin A., Valverde R.A. (2011). Bell Pepper Endornavirus: Molecular and Biological Properties, and Occurrence in the Genus Capsicum. J. Gen. Virol..

[18] Vainio E.J., Chiba S., Ghabrial S.A., Maiss E., Roossinck M., Sabanadzovic S., Suzuki N., Xie J., Nibert M., ICTV Report Consortium (2018). ICTV Virus Taxonomy Profile: Partitiviridae. J. Gen. Virol..

[19] Nibert M.L., Ghabrial S.A., Maiss E., Lesker T., Vainio E.J., Jiang D., Suzuki N. (2014). Taxonomic Reorganization of Family Partitiviridae and Other Recent Progress in Partitivirus Research. Virus Res..

[20] Sabanadzovic S., Valverde R.A. (2011). Properties and Detection of Two Cryptoviruses from Pepper (*Capsicum annuum*). Virus Genes.

[21] Roossinck M.J., Witzany G. (2012). Persistent Plant Viruses: Molecular Hitchhikers or Epigenetic Elements?. Viruses: Essential Agents of Life.

[22] Nemes K., Salánki K. (2020). A Multiplex RT-PCR Assay for the Simultaneous Detection of Prevalent Viruses Infecting Pepper (*Capsicum annuum* L.). J. Virol. Methods.

[23] Kumar S., Stecher G., Suleski M., Sanderford M., Sharma S., Tamura K. (2024). MEGA12: Molecular Evolutionary Genetic Analysis Version 12 for Adaptive and Green Computing. Mol. Biol. Evol..

[24] Matsushita Y., Takeyama S., Tomitaka Y., Matsuyama M., Ishibashi K., Shinosaka H., Osaki K., Kubota K. (2024). Elucidating the Nature of Seed-Borne Transmission of Tomato Brown Rugose Fruit Virus in Tomato, Bell Pepper, and Eggplant. J. Gen. Plant Pathol..

[25] Tollenaere C., Susi H., Laine A.-L. (2016). Evolutionary and Epidemiological Implications of Multiple Infection in Plants. Trends Plant Sci..

[26] Jeger M., Hamelin F., Cunniffe N. (2023). Emerging Themes and Approaches in Plant Virus Epidemiology. Phytopathology.

[27] Roossinck M.J. (2015). Plants, Viruses and the Environment: Ecology and Mutualism. Virology.

[28] Maina S., Donovan N.J., Plett K., Bogema D., Rodoni B.C. (2024). High-Throughput Sequencing for Plant Virology Diagnostics and Its Potential in Plant Health Certification. Front. Hortic..

[29] Babaie G., Izadpanah K. (2003). Vector Transmission of Eggplant Mottled Dwarf Virus in Iran. J. Phytopathol..

[30] Stradis A.D., Parrella G., Vovlas C., Ragozzino A. (2008). Vein Yellowing of Hibiscus Rosa-Sinensis Caused by Eggplant Mottled Dwarf Virus in Southern Italy. J. Plant Pathol..

[31] Wang S., Chen B., Ni S., Liang Y., Li Z. (2024). Efficient Generation of Recombinant Eggplant Mottled Dwarf Virus and Expression of Foreign Proteins in Solanaceous Hosts. Virology.

[32] Bleicher K., Markó V., Orosz A. (2006). Species Composition of Cicada (Auchenorrhyncha) Communities in Apple and Pear Orchards in Hungary. Acta Phytopathol. Entomol. Hung..

[33] Dér Z., Pénzes B., Orosz A. (2003). The Leafhopper Fauna of an Apricot Orchard in Hungary. Acta Phytopathol. Entomol. Hung..

[34] Sándor K., András O. (2024). Adatok a Csepeli-Síkság Kabócafaunájának Ismeretéhez (Hemiptera: Auchenorrhyncha). Biodata Hung. Magy. Biodiverzitás-Kut. Társaság Folyóirata.

[35] Lockhart B.E., Dahal G., Menke J., Olszewski N.E. (2000). Characterization and Genomic Analysis of Tobacco Vein Clearing Virus, a Plant Pararetrovirus That Is Transmitted Vertically and Related to Sequences Integrated in the Host Genome. J. Gen. Virol..

